# Comparative transcriptomic characterization of aluminum, sodium chloride, cadmium and copper rhizotoxicities in *Arabidopsis thaliana*

**DOI:** 10.1186/1471-2229-9-32

**Published:** 2009-03-23

**Authors:** Cheng-Ri Zhao, Takashi Ikka, Yoshiharu Sawaki, Yuriko Kobayashi, Yuji Suzuki, Takashi Hibino, Shigeru Sato, Nozomu Sakurai, Daisuke Shibata, Hiroyuki Koyama

**Affiliations:** 1 Faculty of Applied Biological Sciences, Gifu University, 1-1 Yanagido, Gifu, 501-1193, Japan; 2 Forest Research Institute, Oji Paper Company, 24-9 Nobono, Kameyama, Mie, 519-0212, Japan; 3 BioResource Center, RIKEN, 3-1-1 Koyadai, Tsukuba, Ibaraki, 305-0074, Japan; 4 Laboratory of Plant Environmental Responses, Graduate School of Agricultural Science, Tohoku University, 1-1 Tsutumidori Amamiyamachi, Aoba-ku, Sendai, 985-8555, Japan; 5 Laboratory of Genome Biotechnology, Kazusa DNA Research Institute, 2-6-7 Kamatari, Kisarazu, Chiba, 292-0818, Japan

## Abstract

**Background:**

Rhizotoxic ions in problem soils inhibit nutrient and water acquisition by roots, which in turn leads to reduced crop yields. Previous studies on the effects of rhizotoxic ions on root growth and physiological functions suggested that some mechanisms were common to all rhizotoxins, while others were more specific. To understand this complex system, we performed comparative transcriptomic analysis with various rhizotoxic ions, followed by bioinformatics analysis, in the model plant *Arabidopsis thaliana*.

**Results:**

Roots of *Arabidopsis *were treated with the major rhizotoxic stressors, aluminum (Al) ions, cadmium (Cd) ions, copper (Cu) ions and sodium (NaCl) chloride, and the gene expression responses were analyzed by DNA array technology. The top 2.5% of genes whose expression was most increased by each stressor were compared with identify common and specific gene expression responses induced by these stressors. A number of genes encoding glutathione-S-transferases, peroxidases, Ca-binding proteins and a trehalose-synthesizing enzyme were induced by all stressors. In contrast, gene ontological categorization identified sets of genes uniquely induced by each stressor, with distinct patterns of biological processes and molecular function. These contained known resistance genes for each stressor, such as *AtALMT1 *(encoding Al-activated malate transporter) in the Al-specific group and *DREB *(encoding dehydration responsive element binding protein) in the NaCl-specific group. These gene groups are likely to reflect the common and differential cellular responses and the induction of defense systems in response to each ion. We also identified co-expressed gene groups specific to rhizotoxic ions, which might aid further detailed investigation of the response mechanisms.

**Conclusion:**

In order to understand the complex responses of roots to rhizotoxic ions, we performed comparative transcriptomic analysis followed by bioinformatics characterization. Our analyses revealed that both general and specific genes were induced in *Arabidopsis *roots exposed to various rhizotoxic ions. Several defense systems, such as the production of reactive oxygen species and disturbance of Ca homeostasis, were triggered by all stressors, while specific defense genes were also induced by individual stressors. Similar studies in different plant species could help to clarify the resistance mechanisms at the molecular level to provide information that can be utilized for marker-assisted selection.

## Background

Poor root growth is caused by various rhizotoxic factors present in problem soils, and is linked to susceptibility to other stress factors. For example, aluminum (Al) ions cause severe damage to the roots of plants growing in acid soil, accentuating nutrient deficiency and increasing their sensitivity to drought stress [1]. Other metal rhizotoxins, such as cadmium (Cd) and copper (Cu) ions, also inhibit root growth [2]. The poor development of roots occurs because Al, sodium (Na) and Cu ions have negative impacts on the shoot yield of crop plants in problem soils, while Cd ions decrease the efficiency of phytoremediation in Cd-contaminated soils. Improving the tolerance of roots to rhizotoxic ions is therefore an important target in plant breeding. Understanding of the molecular responses of plants to rhizotoxic ions is a critical step towards molecular breeding of stress tolerant crops using marker-assisted selection or genetic engineering.

Several critical genes regulating tolerance to rhizotoxic ions have been identified in studies using hypersensitive mutants. Studies with salt overly sensitive (SOS) mutants identified genes encoding proteins critical for salt sensitivity, including the Na^+^/H^+ ^antiporter (SOS1) [3] and its regulating protein kinase, SOS2 [4]. Using the Cd- and Al-sensitive mutants, *cad *and *als*, revealed that genes for phytochelatin synthase (*CAD1*) [5] and a putative ATP-binding Al-translocator (*ALS3*) [6] were involved in tolerance mechanisms to these ions. The identification of stress-responsive genes is a useful approach, because some stress-inducible genes might also be involved in tolerance mechanisms associated with abiotic rhizotoxins. For example, the *cis*-element DRE [7], and its binding protein DREB, were identified from a series of studies on dehydration-inducible genes. Several Al-tolerant genes are also responsive to Al ions, such as *ALS3 *[6], *GST *[8] and *AtALMT1 *[9]. Analyses of those genes that are responsive to individual rhizotoxic treatments could also improve our knowledge of the mechanisms of toxicity of the different ions.

Genome-wide transcript analysis can be performed in *Arabidopsis *and other plant species using commercially available oligo-microarray techniques. These techniques have recently been applied to the identification of rhizotoxin-responsive genes in *Arabidopsis *(e.g. NaCl [10] and Al [11]) and other plant species (e.g. Al in *maize *[12,13] and *Medicago *[14]). Those studies demonstrated that various genes were induced by each rhizotoxin. In order to understand the functions and impacts of such gene expression responses to each rhizotoxin, it is important to distinguish those genes induced as part of the general stress responses from those specific to individual stressors. The comparison of transcriptomes among different treatments and the application of bioinformatics procedures (e.g. co-expression gene analysis) are potentially useful approaches for determining the characteristics of these different gene groups.

In order to determine the effects of rhizotoxic treatments on gene expression in *Arabidopsis *using this microarray approach, it is necessary to minimize the effects of other factors on gene expression during the course of the experiment. For example, mechanical damage to the roots triggers the expression of "general" stress-responsive genes [15], and may lead to false conclusions if such a "general response" is not involved in each stress treatment. We previously developed a hydroponic culture system that enhanced rhizotoxicity while minimizing mechanical damage when changing culture solutions [16,17]. This method has been applied to quantitative trait locus analysis of rhizotoxicities [18] and for monitoring root tip viability [19], suggesting that it would also be suitable for obtaining root samples to determine the direct effects of rhizotoxins using microarray analyses. We have also developed an RNA extraction method for *Arabidopsis *that allows the isolation of high quality RNA from various tissues, including roots, at different developmental stages [20]. This can be adapted to rhizotoxin-damaged roots, allowing the isolation of RNA of sufficiently high quality to allow the determination of the complex patterns of gene expression in response to rhizotoxins, using DNA microarray technology.

In the present study, we combined these experimental procedures to analyze gene expression responses in roots by microarray analysis, following treatment with Al, Cu and Cd ions, or NaCl. By comparing microarray data, we were able to separate the general (i.e. common to all rhizotoxic ions) and specific (i.e. more specific to each ion) gene expression responses that were induced by each rhizotoxic ion. Analyses of the separated gene groups based on *Arabidopsis *gene information and bioinformatics tools revealed that both general and individual toxic mechanisms and defense responses were triggered by each rhizotoxic ion.

## Results

### Identification of genes responsive to all ions and to individual rhizotoxic ions

**Figure 1:**
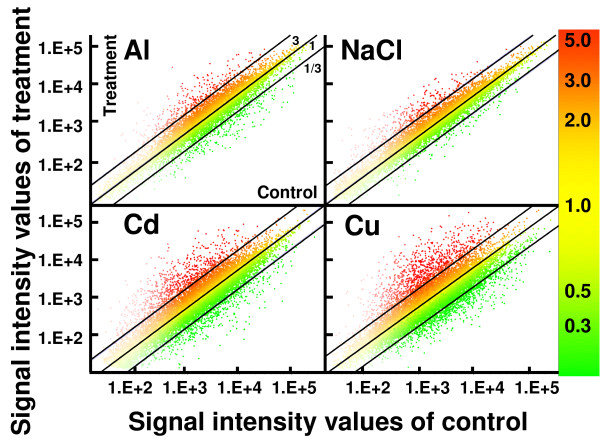
**Scatter plot of competitive microarray data from roots of *Arabidopsis *subjected to rhizotoxins**. Roots of hydroponically grown seedlings were transferred to control (pH 5.0, no toxicant) and rhizotoxic solutions containing AlCl_3 _(25 μM), NaCl (50 mM), CdCl_2 _(15 μM) or CuCl_2 _(1.6 μM) at pH 4.95 (Al) or 5.0 (Others). After 24 h, total RNA was extracted and microarray analyses were performed using the Agilent Arabidopsis 2 Oligo Microarray system. X and Y axes indicate signal intensities in control and rhizotoxic treatments, respectively. Mean of signal intensities from three biologically independent replications are plotted. Fold change (treatment/control) is indicated by color as shown in the color bar in the right side of the panels. Slope of lines in each panel show 3, 1 and 1/3 fold changes, respectively.

**Figure 2:**
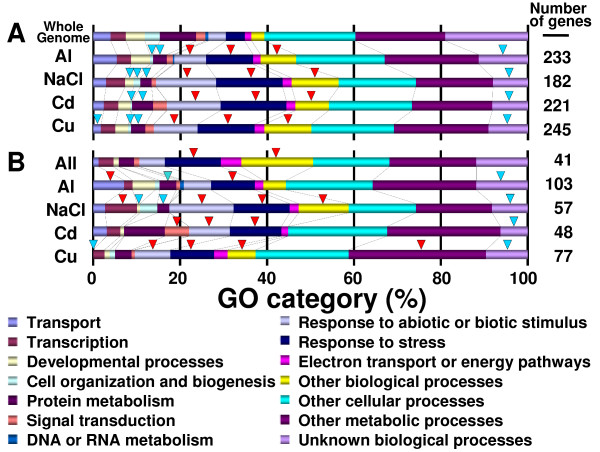
**GO distribution of the gene groups identified by the comparative microarray approach**. Genes highly upregulated by each stressor (A), and those grouped by Venn diagram (B) were classified by GO of biological processes using the TAIR database. (A) Gene groups that were highly induced by each treatment. (B) "All" indicates the gene group overlapped by all ions, while others indicate gene groups uniquely induced by each ion grouped by a Venn diagram (see Figure 3). Genes in the whole genome were also categorized (A). Significance difference from the whole genome was shown with red (higher ratio) or blue (lower ratio) triangles (chi-square test, *P *< 0.05).

The *Arabidopsis *roots grown using the hydroponic culture system were shown by fluorescent probes to be viable (Additional file 1A-a, b). Green color with fluorescein diacetate (FDA) and no visible staining with propidium iodide (PI) indicated that the roots retained esterase activity and integrity of the plasma membrane (Additional file 1B), even after switching the medium. By contrast, the roots were damaged after exposure to rhizotoxic ions (Additional file 2). This indicated that root damage by rhizotoxic treatments was caused by the direct effect of the rhizotoxic ions, and not by artificial mechanical damage. The roots were harvested after exposure to rhizotoxic solutions, and were immediately frozen in liquid N_2 _(Additional file 1A-c, d). This procedure should help to minimize the artificial induction of stress-responsive genes during the experiments. Using this experimental system, we performed microarray analyses after exposure to Al, Cd, and Cu ions, and NaCl (Additional file 3). Although similar levels of stress in terms of the degree of inhibition of root growth were applied (i.e. 90% growth inhibition), Cu and Cd ions induced more genes than Al ions and NaCl (Figure 1). It was difficult to compare genes that were highly upregulated by each treatment if the genes were selected using a single fold change (FC) value as the threshold. Some genes, however, showed large, statistically significant, variations, even if they were repeatedly highly upregulated (Additional file 3). In order to solve these problems, we classified "highly upregulated genes" in each treatment group as those with FC values in the upper 2.5% in each of three independent measurements. These genes were highly upregulated by each rhizotoxic ion, and with high reproducibility. Using this procedure, 233, 181, 221 and 245 genes were identified as being highly upregulated by Al ions, NaCl, Cd and Cu ions, respectively (representing a total of 507 unique genes). Classification of gene ontology (GO) by biological processes showed similar patterns among these "highly upregulated" gene groups, suggesting that all these ions affected various biological events (Figure 2A). However, these gene groups showed distinct GO patterns, compared with those of the whole genome. The gene groups induced by each rhizotoxin contained significantly higher percentages of genes in two categories related to stress responses (i.e. "response to biotic and abiotic stimulus" and "response to stress") and in the category of "other biological processes", relative to the genome as a whole. Conversely, these induced gene groups contained significantly lower percentages of genes attributed to "cell organization and biogenesis", "protein metabolism" and "unknown biological processes" than did the whole genome. These results indicated that our treatments triggered genes responsive to each rhizotoxin.

**Figure 3:**
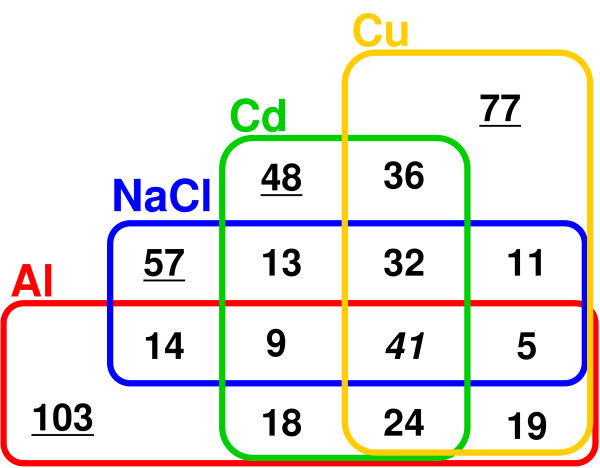
**Venn diagram showing the classification of genes highly upregulated by rhizotoxic ions in *Arabidopsis *roots**. Genes were selected if the fold change value was in the upper 2.5% of quality-controlled spots in each microarray experiment after 24 h incubation with AlCl_3 _(25 μM), NaCl (50 mM), CdCl_2 _(15 μM) or CuSO_4 _(1.6 μM). Genes upregulated in three independent replications were defined as highly upregulated. Genes highly upregulated by each stressor were grouped by Venn diagram. Underlined gene groups consisting of 103 (Al), 57 (NaCl), 48 (Cd) and 77 (Cu) genes were unique for each stressor, while the gene group consisting of 41 genes (italicized) was overlapped by all stressors.

Forty-one genes were co-induced by all ions, while 103, 57, 48 and 77 genes were uniquely identified in the groups of genes highly induced by Al, Cd, and Cu ions, and NaCl, respectively (Figure 3). The common (i.e. overlapped by all four stressors, 41 genes) and the unique gene groups (i.e. unique to one particular stressor) showed different patterns of GO (Figure 2B). For example, the gene groups uniquely grouped by Al ions and NaCl contained significantly higher percentages of genes in the categories related to "transport" and "transcription", respectively. Differences in the gene categories indicated that distinct biological systems might be controlled by the general and specific changes in gene expression caused by rhizotoxic ions. When the genes were categorized by GO for molecular function, different stressors induced distinct gene sets with different molecular functions (Table 1). These differences reflected the character of the gene expression responses of the roots to each rhizotoxic ion.

**Table 1:** Classification by GO categories defined by TAIR for whole genome genes and for gene groups upregulated by rhizotoxic ions identified by a comparative microarray approach.

	**Proportion of genes among GO categories (%)**					
	
**GO slim category**	**Whole Genome**	**All Stressor**	**Al ion**	**NaCl**	**Cd ion**	**Cu ion**
DNA or RNA binding						
hydrolase activity	8.5	14.6	9.7	12.3	10.4	6.5
kinase activity	5.2	2.4	1.0	0.0	16.7**	5.2
nucleic acid binding	4.9	2.4	1.9	1.8	4.2	0.0
nucleotide binding	4.8	2.4	1.9	0.0	8.3	1.3
other binding	13.1	29.3**	16.5	17.5	12.5	16.9
other enzyme activity	10.0	9.8	14.6	15.8	6.3	23.4**
other molecular functions	3.8	4.9	3.9	3.5	4.2	6.5
protein binding	8.5	2.4	7.8	7.0	10.4	6.5
receptor binding or activity	0.9	2.4	1.9	0.0	4.2	0.0
structural molecule activity	2.0	0.0	0.0	0.0	2.1	0.0
transcription factor activity	6.5	7.3	5.8	24.6**	8.3	3.9
Transferase activity	7.5	9.8	16.5**	3.5	6.3	19.5**
Transporter activity	4.8	0.0	10.7**	1.8	8.3	0.0
unknown molecular functions	35.5	22.0	19.4*	26.3	12.5*	20.8

Genes were functionally categorized by GO slim defined by TAIR8. Percentage of the genes attributed to each GO slim category was calculated by the GO annotation tool in the TAIR database. Gene groups were identical to those grouped by Venn diagrams in Figure 3. ** and * indicate that the value in each group is significantly larger or smaller than whole genome, respectively (chi-square test, *P *< 0.05).

### Characteristics of genes induced by all ions

Forty-one genes were identified that responded to all the tested ions (Figure 3; Additional file 3). This group contained a significantly larger percentage of genes with "other binding" activity by GO categorization of molecular function (Table 1), including six Ca-binding proteins, such as calmodulin-like proteins (*CML38 *and *37/39*) and Ca-binding EF hand proteins, which were rare in other gene groups (Additional file 4). Three disease resistance proteins, one belonging to the TIR (Toll-Interleukin-Resistance) class of proteins with molecular transducer activities, were also included in this group, which was previously identified as one of the typical stress responsive genes. The group also contained typical reactive oxygen species (ROS)-responsive genes that encoded ROS-scavenging enzymes (three glutathione transferases and two peroxidases), as well as those involved in the signal transduction pathway for ROS responses, namely *MYB15 *and tolB-related protein. A putative trehalose-phosphate phosphatase gene belonged to this gene group and might be related to the reduction of cellular damage from ROS via the accumulation of trehalose. Induction of these genes could account for the results of previous physiological studies, which reported that ROS production and Ca-alleviation were common features of various rhizotoxicities.

### Characteristics of genes uniquely induced by individual ions

**Figure 4:**
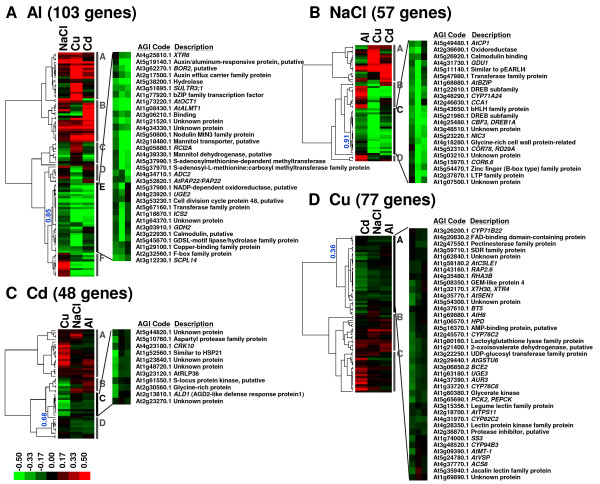
**Hierarchical cluster analyses within gene groups uniquely induced by rhizotoxic ion treatments (*I*_90_)**. Gene groups for Al ion (A), NaCl (B), Cd ion (C) and Cu ion (D) were selected by comparative microarray analysis (Figure 3) and were separately analyzed with a cluster program (see Methods) using the ratio of fold change (FC of other stressor/FC of particular stressor). The ratios of fold change of genes are indicated by color in each panel. Relatively specific clusters are enlarged and the names of genes are indicated for each treatment. Pearson's correlation coefficients were shown in each panel. The enlarged clusters are specific to the stressor than other sub-groups (see Additional file 5).

Venn diagrams demonstrated that some of the genes induced were unique to a particular stressor. These gene groups reflect the toxicity and tolerance mechanisms specific for each ion. The gene group for Al ions contained a known Al-responsive tolerance gene, *AtALMT1 *[9], the Cu ion group contained metallothionein, and the NaCl group included a number of DREB transcription factors, which have been well characterized as key transcription factors regulating NaCl tolerance. On the other hand, those gene groups "unique" to particular stressors included genes that were responsive to other ions, even if these were not included in the upper 2.5%. This indicated that each unique gene group had different characteristics in terms of their specificity to particular ions. We therefore applied cluster analysis to each unique gene group in order to evaluate the specificity of the responses of the genes in these groups to particular stressors (Figure 4). Using relative FC (RFC) values, which were defined as the FC with other stressors relative to that of the particular stressor, we identified specific clusters of genes using hierarchical clustering analysis (Figure 4). The specific clusters for each unique gene group had significantly smaller RFC values than the other clusters (Additional File 5).

#### 1. Genes uniquely induced by Al ions

The Al-responsive group consisted of 103 genes (Figure 3), and included a significantly higher percentage of genes encoding proteins with transporter (10.7%) and transferase (16.5%) activities, by GO categorization of molecular function. Genes encoding transporters were concentrated (i.e. about 19%) in a gene cluster containing 32 genes (Figure 4A), which were relatively specific to Al ions (Table 1). Major transporters for sulfate (*SULTR3;1*) and borate (*BOR2*) were found in this specific cluster, together with *AtALMT1 *and other organic molecule transporters [e.g. mannitol and the organic cation/carnitine transporter (*AtOCT1*)]. This specific gene cluster also contained genes encoding an auxin/Al-responsive protein, an auxin carrier protein, and a gene encoding purple acid phosphatase.

Although genes encoding transferases were not concentrated in a specific gene cluster, two S-adenosyl-L-methionine:carboxyl methyltransferase family proteins and three carbohydrate transferases (e.g. glycosyltransferase) belonged to this gene group. A large number of genes involved in carbon and nitrogen metabolism were also identified in this Al-specific group, including glutamate dehydrogenase (*GDH2*), malic enzymes (*AtNADP-ME1 *and *2*), and some carbohydrate decarboxylases, including a pyruvate decarboxylase (Additional file 3).

#### 2. Genes uniquely induced by NaCl

Venn diagram analysis identified 57 genes that were uniquely induced by NaCl treatment (Figure 3). GO analysis for molecular function suggested that this gene group contained a significantly higher percentage of genes encoding transcription factors (24.6%) (Table 1), while GO analysis for biological process found a higher percentage of genes in the transcription category (Figure 2). This group contained more transcription factors, including some DREB family proteins (three of a total of six DREB families identified in all gene groups), which have been recognized as playing a role in salt tolerance. Cluster analysis revealed that 22 genes, including seven transcription factors, were more specific to NaCl than were the other genes (Figure 4B). Some cold-responsive genes (e.g. *COR6.6*, *COR78*), whose signal transduction pathways overlap with NaCl stress, were also identified in this cluster. No genes for major catalytic enzymes involved in carbon or nitrogen metabolism, and only one transporter, were found in the NaCl group.

#### 3. Genes uniquely induced by Cd ions

The Cd ion-induced gene group contained no catalytic enzymes involved in major primary or secondary metabolism, but did include some protein kinases, such as receptor-like protein kinases (*CRK6 *and *10*) [21] (Additional file 3). This could account for the significantly higher ratio of genes with "kinase activity" (16.7%), when genes were categorized by molecular function (Table 1). Genes belonging to the enriched GO category were not enriched in the specific gene cluster (Figure 4C). The specific cluster, also, contained several stress-responsive genes, whose functions such as heat-shock and defense-response, have not yet been clarified (Figure 4C). One gene categorized by GO as having kinase activity, a leucine-rich repeat family protein (*AtRLP38*) similar to disease resistant proteins, was also identified in this specific gene cluster.

#### 4. Genes uniquely induced by Cu ions

The Cu ion group contained known Cu-detoxifying and binding molecules, such as metallothionein (*MT2A*) (Additional file 3). A large number of secondary metabolite-synthesizing enzymes involved in "other metabolic processes" (Figure 2), such as strictosidine synthase 3 (*SS3*) (involved in alkaloid synthesis), anthranilate synthase and six isoforms of cytochrome P450 were also identified in this group. These could account for the significantly higher percentages of genes encoding proteins with other enzyme activities (23.4%) and transferase activities (19.5%) (Table 1). Two trehalose synthases (*ATTPS8 *and *11*) and a ROS-scavenging protein, namely thioredoxin H-8 (*ATH-8*), may reflect the relative severity of ROS production induced by Cu ion treatment, compared with the other ions. In the Cu ion-specific gene cluster, an l-aminocyclopropane-1-carboxylate synthase (ACC synthase; *ACS8*) belonging to the ethylene biosynthesis pathway was identified, together with an enzyme relating to auxin synthesis [i.e. an indoleacetic acid (IAA) amide synthase (*AUR3*)]. An enzyme synthesizing the precursor of IAA, tryptophan, namely tryptophan synthase alpha chain (*TSA1*) and beta chain (*TSB1*), were identified in the Cu ion-responsive gene group.

### Root tip viability, cell damage and ROS production following rhizotoxic treatments

**Figure 5:**
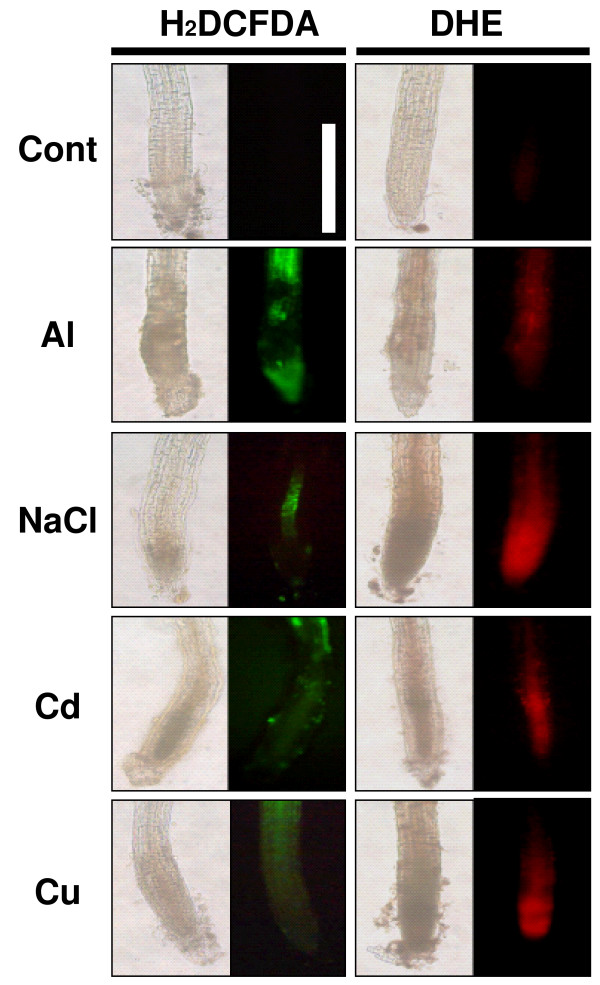
**Histochemical analyses of roots of *Arabidopsis thaliana *after incubation in rhizotoxic solutions**. Growing roots were immersed in rhizotoxic solutions containing AlCl_3 _(25 μM), NaCl (50 mM), CdCl_2 _(15 μM) or CuSO_4 _(1.6 μM) for 24 h, stained with 2',7'-dichlorodihydrofluorescein diacetate (H_2_DCFDA) or dihydroethidium (DHE), and then observed under a fluorescence microscope. Fluorescent and bright field images are shown. Images of non-stressed roots are shown as controls. White bar indicates 100 μm.

The induction of ROS-scavenging enzymes in the shared gene group indicated that all stressors caused an accumulation of ROS. To confirm this possibility, the roots were stained using fluorescent probes to detect hydrogen peroxide (H_2_O_2_) (i.e. 2',7'-dichlorofluorescein diacetate, H_2_DCFDA) and superoxide anions (O_2_^-^) (i.e. dihydroethidium, DHE), respectively. In all four treatments, green and red fluorescence were generated by H_2_DCFDA and DHE, respectively, while the roots in control preparations (without stressor) showed no visible fluorescence (Figure 5). Although the intensity of staining in the roots treated with stressors may not directly reflect the level of ROS production, because of a metal-quenching effect during fluorescent staining, these results indicated that ROS were induced by all stressors, but with different patterns (i.e. different locations in the root tissue and different ROS species). The gene group shared by all stressors contained a large number of ROS-scavenging enzymes, while the unique groups contained additional ROS-scavenging enzymes that could account for the different staining patterns seen with different treatments (Additional file 4).

### Co-expression gene analysis within each group

**Figure 6:**
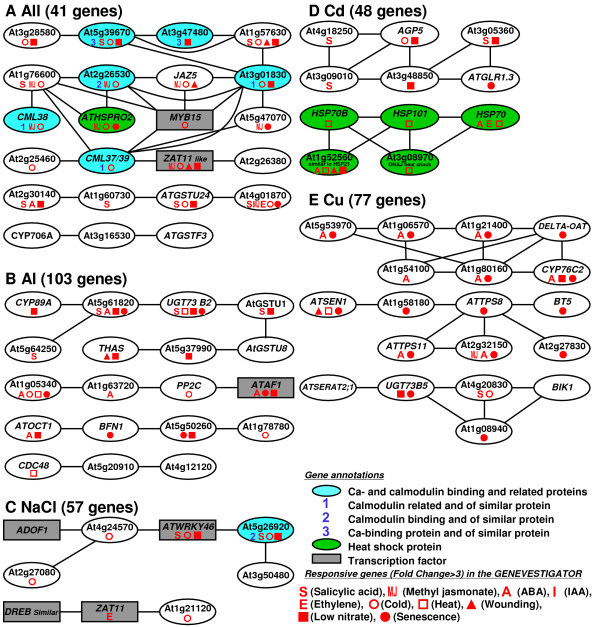
**Co-expressed genes network within the gene groups identified by comparative microarray approach (see Figure 3)**. Gene groups responsive to all tested rhizotoxins (Al, NaCl, Cd and Cu) and those uniquely induced by each stressor were analyzed to identify co-expressed gene networks by KAGIANA software, using a co-expression gene data set available in the ATTED-II database. Gene clusters were connected with lines if their Pearson's coefficient of correlation for gene expression was > 0.6 among 1388 microarrays from 58 experiments, which are available in the TAIR database. Other detailed information can be seen on the KAGIANA web site . Some of genes in the cluster are colored according to their molecular functional annotations, and the characteristics of gene expression response reported by GENEVESTIGATOR are shown with various symbols (see low right of the Figure).

Co-expression gene analysis was carried out using KAGIANA software, which allows for the identification of co-expressed genes among gene groups, based on correlation coefficients from publicly available microarray data derived from the ATTED-II database (see detail at ATTED-II web site; ). One large cluster consisting of 16 genes was identified in the gene group that overlapped for all stressors (Figure 6A). This group contained a number of Ca-binding proteins (calmodulin and its related proteins) and transcription factors (*MYB15 *and an unidentified member of the ZAT (*ZAT11 *similar) zinc finger protein containing an EAR repressor domain). Response viewer in the GENEVESTIGATOR showed that this gene group also responded to other biotic and abiotic stressors, such as ozone, nematodes, H_2_O_2 _and AgNO_3 _(Additional file 6), suggesting that these genes were commonly responsive to various stress treatments. One cluster in the shared gene group contained four genes that were responsive to salicylic acid (Figure 6A). For each individual treatment, 2–4 clusters were identified by the same analyses (Figure 6B–E). The NaCl-responsive genes formed two clusters containing a homolog of DREB (Figure 6C), and cold-responsive genes. One of two clusters in the Cd-responsive group consisted of genes upregulated by heat treatment, while the other cluster showed no response to heat treatment (Figure 6D). Two clusters in the Cu-specific group contained genes responsive to senescence, one of which was also responsive to abscisic acid (ABA) (Figure 6E). These analyses indicated that distinct gene expression networks were triggered by each stressor, while some networks were shared by all stressors.

## Discussion

**Figure 7:**
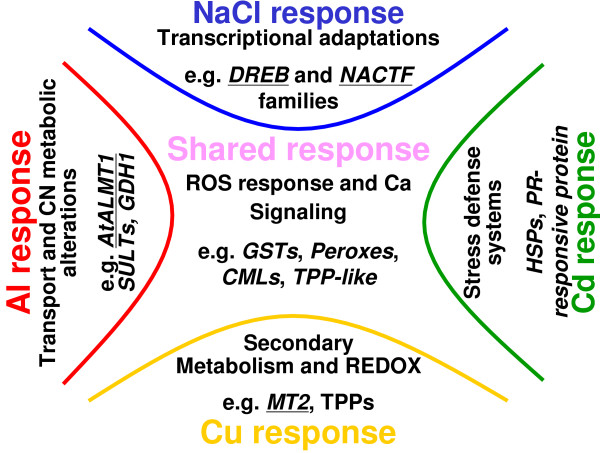
**Schematic representation of genes responsive to rhizotoxic ions, as identified using comparative microarray analysis**. Typical responsive genes induced by all ions, and those induced by individual ion treatments are shown. Genes previously identified as critical for stress tolerance are underlined.

Rhizotoxicity studies based on the inhibition of root elongation caused by the ionic activity of toxins at the plasma membrane surface, have indicated that different ions exert distinct toxic actions [22], but also that almost all ions stimulate some common stress-responsive processes, such as ROS production and enhanced secondary metabolism [23,24]. To induce all toxin-responsive genes, we employed relatively higher concentrations of rhizotoxic ions than those required to inhibit root elongation, though these treatments also reduced root viability (Additional file 2), suggesting that our treatments triggered genes involved both in defense systems and in damage response. Changes in gene expression caused by toxic ions might therefore reflect these complex factors. By comparing microarray data between different treatments, we identified gene groups induced as part of general stress responses, as well as those specifically induced in response to individual toxic ions (Table 1, Additional file 3). These gene groups agreed with the results of histochemical observations (Figure 5) and with the functions of some genes previously identified in other molecular biological studies (Figure 7).

The group of genes that was responsive to all ions contained a large number of genes encoding ROS-scavenging enzymes, such as glutathione transferase and peroxidases, and an enzyme for producing trehalose, whose accumulation stabilizes cellular structure against ROS damage [25]. Overexpression of these genes conferred abiotic stress tolerance [26-28] and their induction would therefore act as part of the defense responses against ROS damage induced by all stressors. One large cluster of genes in this group, identified by co-expression gene analysis by KAGIANA search (see Methods), contained various Ca-binding proteins, including previously identified calmodulin-like proteins (*CML37/39 *and *38*), which were inducible by various stimuli [29], suggesting that Ca-mediated signaling pathways could play important roles in the *Arabidopsis *response to rhizotoxic stressors. A previously identified transcription factor *MYB15*, which is involved in the cold stress-mediated defense system associated with *ICE1 *(inducer of CBF expression) [30], was also included in this cluster. It seems likely that this gene group, which was responsive to all ions, is related to the general stress-responsive system in plants. Although the pattern of staining was different, ROS accumulation occurred in the roots subjected to milder rhizotoxic conditions (i.e. concentrations causing 50% growth inhibition) (Additional file 7). This suggests that ROS production is a general feature of rhizotoxic treatments. It is interesting to note that the induction of ROS-scavenging enzymes was common to all stressors, and occurred even under mild stress conditions.

The gene groups responsive to individual ions included those genes typically upregulated by each stressor. For example,*AtALMT1 *was highly upregulated by Al ions, but was not responsive to other ions [31]. In addition, the upregulation of this gene was the largest detected among all the genes (Additional file 3), suggesting that it plays a critical role in the active Al ion defense system of this plant species [9]. Interestingly, the bypass pathways of tricarboxylic acid and glutamate metabolism were also relatively upregulated by Al ion treatment, compared with other treatments (Additional file 3). This could be related to malate efflux, because organic acid excretion can be enhanced by transgenic modification of several enzymes involved in tricarboxylic acid metabolism and its bypass (e.g. citrate synthase [32]), though the regulation of cytosolic pH caused by changes in these bypass pathways is a possible alternative mechanism. These possibilities need to be tested by future research.

Other rhizotoxic ions, namely NaCl, Cd and Cu ions, induced distinct and specific sets of genes (Figure 3, Additional file 3). For example, gene clusters in the Cu ion-responsive group consisted of senescence-responsive genes, including a gene encoding a previously identified senescence related protein (*AtSEN1*), which enhances mRNA degradation [33]. This may be related to the stimulation of secondary metabolism pathways, such as terpenoid indole alkaloid metabolism, involving strictosidine synthases (*SS2*; [34]) and tryptophan synthases (*TSB2 *[35]; *TSA1 *[36]), which are activated in mature and senescent tissues. The Cu ion-responsive group also contained various defensive genes, such as *ATTPS8 *and *11 *[37], which are involved in trehalose synthesis, in addition to the well-characterized Cu ion-detoxifying protein metallothionein (*MT2*), indicating enhancement of ROS-scavenging capacity. Cu treatment also stimulated thioredoxin gene expression (thioredoxin H-8 (*ATH-8*) [38]), which is involved in the Cu ion tolerance mechanism of some organisms [39]. The *Salmonella *thioredoxin homolog possibly acts by reducing free Cu ions through regulating the binding capacity of the reduced form of thioredoxin to Cu ions [40]. Taken together, the Cu ion group contained genes reflecting the toxicity of Cu and defensive genes that produced proteins to alleviate Cu toxicity.

The other uniquely identified gene groups had similar compositions. The NaCl group demonstrated the importance of the DREB system in defense [41]. Although previous studies have reported that the *DREB1A *family was responsive to cold treatment, but not to Na ions [41], our data indicate that this family is also involved in the NaCl-responsive system in the root. This discrepancy might be because of differences in strength of the NaCl used, as our treatment was almost five times milder (50 mM) than that used in previous molecular biological studies (e.g. [41]). On the other hand, the Cd ion-responsive gene group consisted of unidentified stress-responsive proteins, which were categorized as heat shock and pathogen-related proteins. Further research is needed to clarify the role of these proteins in Cd tolerance.

When we applied the same experimental design using the lower 2.5 percentile as the threshold, we are able to characterize the groups of genes downregulated by each stressor (Additional files 8, 9). GO annotation by molecular function (Additional file 10) showed that uniquely identified groups of genes had distinct patterns. For example, genes with "hydrase activity" were increased by NaCl, or Cd and Cu ions, while those for "transporters" were increased by Al ion treatment (Additional file 9). On the other hand, several genes relating to defense responses, such as disease-resistance related protein, were found to be downregulated by all stressors. This suggests that a combination of up-regulation and downregulation of stress responsive genes may be important in optimizing the adaptation of particular biological pathways to stress conditions.

Co-expressed gene clusters may reflect the cellular conditions and activated defense systems induced by each stressor. For example, Al ions induce phosphate deficiency as a secondary effect [1], while defense systems for abiotic stressors are activated by phytohormones (e.g. ABA in Cd and Na tolerance [42]). Based on the upregulations recorded by GENEVESTIGATOR [43], we may infer that the ABA signaling pathway was activated by both Cu and Al treatments, because a large portion of one cluster in both the Cu ion- (6/7 in the upper cluster; Figure 6E) and Al ion- (3/4 in the middle cluster; Figure 6B) responsive groups consisted of ABA-responsive genes. Furthermore, activation of the salicylic acid signaling pathway was involved in the responses to all treatments, because a cluster responsive to salicylic acid was identified in the shared gene group. These results could explain the involvement of these signaling pathways in the tolerance mechanisms for each stressor (e.g. ABA signal in Al [44] and Cu tolerance [45]; salicylic acid signal in Al [46], NaCl [47], Cd [48] and Cu tolerance [49]).

To investigate the changes in gene expression caused by various rhizotoxic ions, we employed a simple experimental design using a limited number of microarrays (i.e. single time point and single treatment for each ion). This could be advantageous in terms of experimental costs when applying a similar approach to other plant species. Accurate information (e.g. GO) provided by recent developments in the functional genomics of *Arabidopsis*, is critically important for the success of this approach. Similar developments in genomic research are becoming available for other plant species, and we can therefore apply this procedure to other plant species, and can use comparative genomics to compare the resistance (and damage) systems to rhizotoxic ions among different plant species. Integrated analyses with other -omics data (e.g. metabolomics) would also be interesting to further our understanding of tolerance to and toxicity of rhizotoxic stressors.

There are limitations to our current approach, and several questions remain. For example, we focused on the genes upregulated either collectively or specifically by four different ions. This method excluded genes that were upregulated by two or three stressors, though they may also play an important role in defense and stress-response. For example, some genes encoding cell wall-associated proteins and vacuole loading proteins, which are known to be involved in Cd and Al tolerance, were excluded by our approach. On the other hand, we selected upregulated genes using the upper 2.5 percentile as a threshold. This relative threshold value was preferable to using an absolute fold change threshold value, allowing the selection of a similar number of genes from each treatment group, despite variable distributions of fold changes. This allowed comparison among the groups of genes with similar weights of importance. However, our procedure cut-off the genes if their fold change values were just below the upper 2.5 percentile. The impact of these genes would therefore have been underestimated by the present analysis. Further investigation of these genes using a different method of data analysis is required for a complete understanding of the complex nature of rhizotoxicities.

## Conclusion

Using genome-wide DNA microarray technology, we analyzed the impact of rhizotoxic ions (Al, Cd and Cu) and NaCl on gene expression in the roots of *Arabidopsis*. Comparison of the microarray data allowed the induced genes to be grouped into those common to all treatments, and those unique to individual treatments. Each gene group contained reported tolerance genes, such as *AtALMT1 *in Al treatment, *DREB *in NaCl treatment and *MT2 *in Cu treatment. ROS-scavenging enzymes and Ca-binding proteins, however, were in the group of genes that was upregulated by all stressors. These results were consistent with tolerance mechanisms identified in previous physiological studies. In addition, bioinformatics analyses of the genes groups showed that distinct physiological responses were induced by each stressor. Overall, we showed that comparative microarray analysis with a simple experimental design was a useful technique for identifying gene responses that were consistent with the cytotoxic and tolerance mechanisms of the roots to rhizotoxic ions. Further data analysis, such as a comparison of downregulated genes and the integration of other -omics based technologies (e.g. metabolomics) would be useful for further research into the complex nature of the responses of plant roots to rhizotoxic stressors. Recent developments in genomic research in other plant species may allow us to use similar approaches in various plant species, allowing useful comparisons to be made regarding the similarities and differences in the tolerance mechanisms among different plant species.

## Methods

### Rhizotoxic treatments

Accession Col-4 (N933, NASC; Nottingham Arabidopsis Stock Center) seedlings were pre-grown for 10 days in modified MGRL medium (pH 5.0), as described previously [17] (2% MGRL nutrients, but the with the Ca ion concentration adjusted to 200 μM) The culture apparatus was made from plastic and resin fibers, which were stable in the presence of the chemical rhizotoxins [16] (Additional file 1). About 200 seedlings were grown in each culture apparatus. At day 10, the seedlings were transferred, together with the culture apparatus, to 500 ml of modified MGRL medium, containing either 25 μM AlCl_3 _(pH 4.95), 50 mM NaCl (pH 5.0), 15 μM CdCl_2 _(pH 5.0) or 1.6 μM CuSO_4 _(pH 5.0). In all cases, appropriate stock solutions were used to minimize precipitation and change of ionic form. Two liters of each toxic solution was used for the treatment of 200 seedlings. Under these conditions, the pH of the solution was stable (ΔpH < 0.03) and the toxic ions remained soluble (i.e. concentration in the supernatant fractions obtained by centrifugation at 20,000 g, for 15 min) after 24 h incubation. Gene expression could be influenced by secondary effects (e.g. apoptosis or necrosis) if the treatment was too severe, whilst too weak treatment might not trigger the expression of some of the rhizotoxin-sensitive genes. As a compromise, rhizotoxic treatments were carried out using concentrations of Al, Cd, and Cu ions, and NaCl that caused approximately 90% growth inhibition during a 1-week growth test (data not shown). Control pre-grown seedlings were transferred to the basal test solution on day 10 (no stress). Room temperature was maintained at 23–25°C and illumination was controlled at 12 h daytime (30 μmol E m^-2 ^s^-1^)/night time (no illumination) cycles during pre-growth, and continuous illumination during rhizotoxic treatments. After treatment with rhizotoxins for 24 h, seedlings were removed from the apparatus using forceps. Roots were then rinsed in distilled water and excess water was removed by absorption with tissue. Roots were excised with scissors, immediately frozen in liquid N_2 _and stored at -80°C in plastic sample tubes (5 ml) until use (Additional file 1A–c, d).

### RNA isolation

Total RNA was isolated using the method described by Suzuki et al. [20] then quantified at A_260, _using a NanoDrop spectrophotometer (ND-1000, NanoDrop Technologies, Wilmington, DE, USA). The quality of RNA used for microarray analysis was measured using an Agilent 2100 Bioanalyzer (Agilent Technologies, Palo Alto, CA, USA), according to the manufacturer's instructions.

### Microarray experiment

Microarray analyses were carried out using a competitive hybridization method (i.e. dye-flip method) using the Agilent microarray system (Agilent Technologies, Palo Alto, CA, USA). All procedures were carried out according to the manufacturer's protocols. Briefly, 1 μg of total RNA from each sample was used to synthesize cRNA and was labeled with cyanine-5 (Cy5)- or cyanine-3 (Cy3)-labeled CTP (Perkin Elmer/NEN Life Sciences, Tokyo, Japan). The labeled cRNAs (including a treatment and a control sample) were competitively hybridized to the Agilent Arabidopsis 2 Oligo Microarray, and then washed. The hybridized slides were scanned using Agilent DNA Microarray Scanner (Software Version 6.1) and data points were extracted using Agilent Feature Extraction software (Version 8.1). Three comparisons, including one dye-flip, were made between biologically independent samples. All microarray data have been deposited in a public database (we will complete this before final acceptance). Expression profile trends for the selected genes were examined by reverse transcription-polymerase chain reaction (RT-PCR) using specific primers. Expression patterns judged by RT-PCR were similar to those examined by microarray analysis (Additional file 11). All microarray data are available through the ARRAYEXPRESS database  with accession code of E-MEXP-1907 (Transcription profiling of Al, Cu, Cd, NaCl, stress).

### Data analysis

Statistical analyses of microarray data and drawing of scatter plots were performed using GeneSpring GX 7.3 (Silicon Genetics, Redwood City, CA, USA), while identification of GO and classification were carried out using software (e.g. ) available from the Arabidopsis Information Resource (TAIR) database . Data points based on less than three measurements and spots with low fluorescence intensity (i.e. 80 <) were excluded from the analyses. After this quality control, 15,523 genes were selected from those spotted on the Agilent Arabidopsis 2 microarray slides. Genes were considered to be highly upregulated by a stressor if their fold change values were in the upper 2.5% (i.e. upper 388 genes, based on fold change values) in all three replications. Based on these criteria, 233, 181, 221 and 245 genes were identified as being highly upregulated by Al, Cd or Cu ions, or NaCl, respectively. The means and standard errors (SE) of log_2 _(fold changes) were calculated for each data point using GeneSpring GX 7.3. Genes upregulated by all stressors and those uniquely responding to each stressor (designated as unique gene groups) were identified by Venn diagrams.

Cluster analyses were performed using CLUSTER software [50] (available at ) to group the genes in the unique gene groups by specificity of their responses to a particular stressor. The mean FC with other ion treatments was divided by the mean FC with a particular stressor (e.g. FCs for NaCl, Cd and Cu treatments in the Al unique group were divided by the FC for Al treatment), and were designated as "relative fold change" (RFC). The RFCs of the unique gene groups were separately introduced to the Cluster software and then grouped with hierarchical clustering using the average linkage clustering method. Data were normalized to the mean RFC for each treatment. The output data were visualized using the TREEVIEW program. Under these conditions, genes that were highly specific to a particular stressor showed as greenish in color. Genes were manually sub-grouped by the formed cluster and its color, and specificity was assessed by comparison of sub-groups using the Scheffe test (*P *< 0.05).

Co-expression gene analysis was performed using KAGIANA software  using a co-expression gene file obtained from ATTED-II [51], which consists of 58 experiments including 1388 arrays, and was downloaded from TAIR. Co-expression gene clusters were connected by lines if their Pearson's coefficients of correlation for gene expression were > 0.6. Expression profiles of the clustered genes to various treatments were collected from the response viewer of the GENEVESTIGATOR database (; see Additional file 6).

### Histochemical analyses

Cell viability, plasma membrane integrity and accumulation of ROS (H_2_O_2 _and O_2_^-^) were visualized using fluorescent probes, as described previously [19,52]. Briefly, growing roots (day 5) were transferred to stress conditions identical to those used for microarray experiments. Cell viability was determined by FDA staining (5 μg/ml for 30 s), which generates green fluorescence in viable cells. Plasma membrane damage was visualized by PI (3 μg/ml for 1 min), while H_2_O_2 _and O_2_^- ^accumulation were visualized using H_2_DCFDA (10 μM for 10 min) and DHE) (10 μM for 30 min, 37°C), respectively. Fluorescence in the root tip was observed using a fluorescence microscope (IMT-2-21-RFL, Olympus, Tokyo) equipped with appropriate dichroic mirror units (PI, IMT-2-DMG; FDA, H_2_DCFDA; DHE, IMT-2-DMIB). Images were photographed using a digital camera unit (PMDC α/OL-1, Olympus). GO was searched for on TAIR using a web tool for GO annotations and categorization [53].

## Authors' contributions

ZC-R, TI, YS, TH, SS and NS participated in the microarray experiments and data analyses. ZC-R and TI participated in the histochemical analyses and qRT-PCR analyses. ZC-R, YK, YS and TI participated in the data analyses. ZC-R, TI, DS and HK wrote the manuscript, and designed research with NS and YK. All authors read and approved the final manuscript.

## Supplementary Material

Additional file 1 **Hydroponic culture and sampling of root tissues of *Arabidopsis***. (A) Seedlings were grown on plastic mesh floated on control solution. Top (a) and side (b) views at 10 days are shown. Roots were excised with scissors (c), immediately frozen in liquid nitrogen (d) and used for RNA isolation and microarray analysis. White bar indicates 10 mm. (B) Viability of the root tip in 10-day-old seedlings grown in the culture apparatus in control solution. Root tips were stained with fluorescein diacetate (FDA) and propidium iodide (PI). Bright field images are also shown. White bar indicates 100 μm. Click here for file

Additional file 2 **Viability of root tips of *Arabidopsis thaliana *under microarray conditions**. Seedlings were incubated for 24 h in rhizotoxic solutions (*I*_90 _level) and then stained with fluorescein diacetate (FDA) and propidium iodide (PI). Bar indicates 100 μm. Red color indicates damage of the plasma membrane due to PI fluorescence, while green fluorescence of FDA visualizes viable cells. Click here for file

Additional file 3 **List of genes up-regulated by rhizotoxic ions and grouped by Venn diagram**. Highly up-regulated genes in Al, Na, Cd and Cu treatments were grouped by Venn diagram as shown in Figure 3. GO annotation and functional category at the TAIR database, and the fold change in microarray experiments are summarized for each gene in the list. Click here for file

Additional file 4 **Grouping of genes encoding ROS-scavenging enzymes and Ca-related proteins among highly inducible genes (i.e. genes grouped in **Figure 3**) using a Venn diagram approach**. (A) Genes encoding ROS-scavenging enzymes, superoxide dismutase, glutathione transferase and peroxidases. (B) Genes encoding proteins carrying "Ca-binding" or "Calmodulin" in their annotation. Relative values (% in each category in Figure 3) are also shown. Click here for file

Additional file 5 **Mean of the relative fold change of the genes that were grouped by cluster analysis in **Figure 4. Mean of relative fold change of the genes belongs to specific gene cluster was statistically compared to those of others. The mean of specific clusters, the cluster E of Al unique genes group and the clusters C of NaCl, Cd or Cu unique genes groups, were same or significantly less than other clustered gene groups when judged by Scheffe test (*P *< 0.05). Click here for file

Additional file 6 **Networked genes within the uniquely identified gene groups, and their response to various treatments summarized by response viewer of GENEVESTIGATOR**. By coexpression gene analysis, the networked genes groups were identified within uniquely identified genes groups by a Venn diagram approach in Figure 3 (see Figure 6). Fold change values of the networked genes to various stress treatments were manually collected from the response viewer of "GENEVESTIGATOR" through the TAIR database. Click here for file

Additional file 7 **Histochemical analyses of roots of *Arabidopsis thaliana *after incubation in rhizotoxic solutions (*I*_50_)**. Growing roots were immersed in rhizotoxic solutions containing AlCl_3 _(6 μM), NaCl (10 mM), CdCl_2 _(3 μM) or CuSO_4 _(1.4 μM) for 24 h, stained with 2',7'-dichlorodihydrofluorescein diacetate (H_2_DCFDA) or dihydroethidium (DHE), and then observed under a fluorescence microscope. Fluorescent and bright field images are shown. Images of non-stressed roots are shown as controls. White bar indicates 100 μm. Click here for file

Additional file 8 **List of genes down-regulated by rhizotoxic ions and grouped by Venn diagram**. Highly down-regulated genes by rhizotoxic treatments were grouped by Venn diagram as shown in Additional file 9. The file includes the list of genes with their fold change values, GO annotation and functional category according to the TAIR database (TAIR8). Click here for file

Additional file 9 **Venn diagram showing the classification of genes highly downregulated by rhizotoxic ions in *Arabidopsis *roots**. Genes were selected if the fold change value was in the lower 2.5% of quality-controlled spots in each microarray experiment after 24 h incubation with AlCl_3 _(25 μM), NaCl (50 mM), CdCl_2 _(15 μM) or CuSO_4 _(1.6 μM). Genes downregulated in three independent replications were defined as highly downregulated. Genes highly downregulated by each stressor were grouped by Venn diagram. Underlined gene groups consisting of 81 (Al), 73 (NaCl), 52 (Cd) and 34 (Cu) genes were unique for each stressor, while the gene group consisting of 18 genes (italicized) was overlapped by all stressors. Click here for file

Additional file 10 **Classification by GO categories defined by TAIR for whole genome genes and for gene groups downregulated by rhizotoxic ions identified by a comparative microarray approach**. Genes were functionally categorized by GO slim defined by TAIR8. Percentage of the genes attributed to each GO slim category was calculated by the GO annotation tool in the TAIR database. Gene groups were identical to those grouped by Venn diagrams in Additional file 9. ** and * indicate that the value in each group is significantly larger or smaller than whole genome, respectively (chi-square test, P < 0.05). Click here for file

Additional file 11 **Gel image of amplicons derived from semi-quantitative RT-PCR for selected genes**. Pattern of gene expression profiles of selected genes by Venn diagram (see Figure 3) and cluster analysis (see Figure 4) were analyzed by semi-quantitative RT-PCR. The PCR conditions were optimized to ensure the linear phase of amplification and gel image detection. The *UBQ1 *expression is shown as control of gene expression. Amplicons were separated on a 3% agarose gel and then visualized with the 1×SYBR Green I (Invitrogen, USA). The gel images were captured with an image analysis system (Typhoon 9400, Amersham Biosciences). Total RNA was isolated from biologically independent root samples with the same rhizotoxic treatments (Al, Cd, Cu ions and NaCl) that were used for the microarray analysis. PCR condition and primers were as follows: *AtALMT1 *forward: 5'-GGC CGA CCG TGC TAT ACG AG-3', reverse: 5'-CTG AAG ATG CCC ATT ACT TA-3'(263 bp, 22 cycles); *AtOCT1 *forward: 5'-TTTCTTGTGGCTGTTCCTTCCACAC-3', reverse: 5'-TCT GGA ATT GGA TCG ACT AGG CTT A-3'(548 bp, 23 cycles); *DREB1A *forward: 5'-GAT GTG TGA TGC GAC GAC G-3', reverse: 5'-TCC ACT GTA CGG ACG GAA G-3'(182 bp, 26 cycles); *RD29A *forward: 5'-TTC AGA CTA TCT TAG TGG T-3', reverse: 5'-CGT CAC CAA AGC CCA CCG G-3'(281 bp, 26 cycles); At1g52560 forward: 5'-ATA CGA GGT TCC AGG GCT AAC CAA A-3', reverse: 5'-CAA AAA CGA CAC CGT ATC TCT TCT A-3'(305 bp, 31 cycles); *HSP70 *forward: 5'-TGT ACC AAG GAG CTG GGC CTG ATA T-3', reverse: 5'-GCC CAG TCG TCT TTC ATA GGT CAG A-3'(275 bp, 31 cycles); *AtH8 *forward: 5'-AGG CTC AAC GCT CTT AAA GAC ACC A-3', reverse: 5'-TGA ATA CAA TCG CAG GTA AAG TGC T-3'(205 bp, 28 cycles); *AtGSTU24 *forward: 5'-TCA TTA CAT TCA TTT CCG AAC GTA G-3', reverse: 5'-TTA TTA TGC ATT ACA TAG ACC TCA A-3'(119 bp, 25 cycles); *AtGSTU11 *forward: 5'-TAT CGA AAA ACT GGT CCA GTT CGC T-3', reverse: 5'-CCT TTT AAC TAA ACG AGT TTA CAT C-3'(150 bp, 33 cycles). Click here for file
