# Supplementary figures and images for: Comparative transcriptomic characterization of aluminum, sodium chloride, cadmium and copper rhizotoxicities in Arabidopsis thaliana
Source: BMC Plant Biol. 2009 Mar 23;9:32. doi: 10.1186/1471-2229-9-32 (PMC2666732; doi:10.1186/1471-2229-9-32)

**A**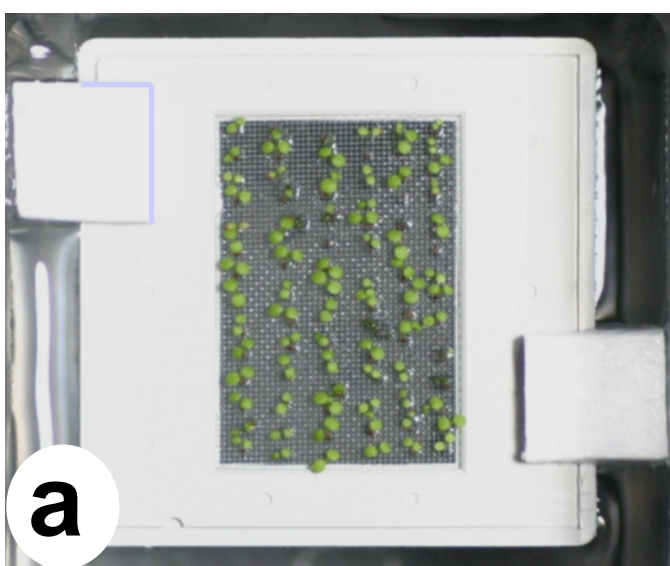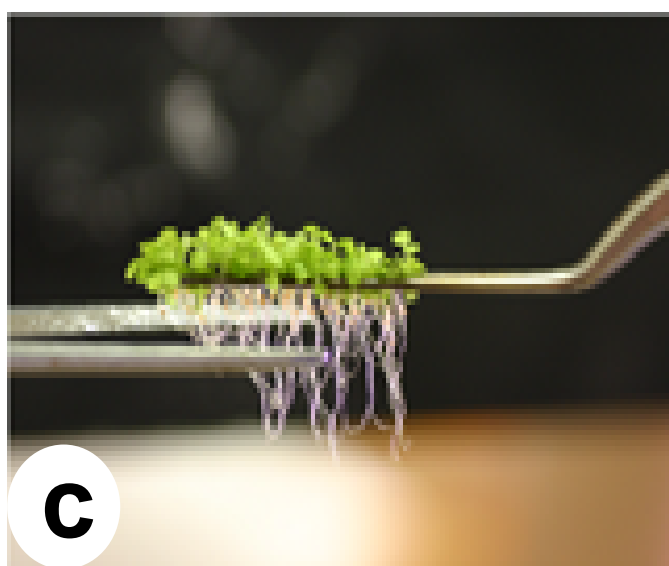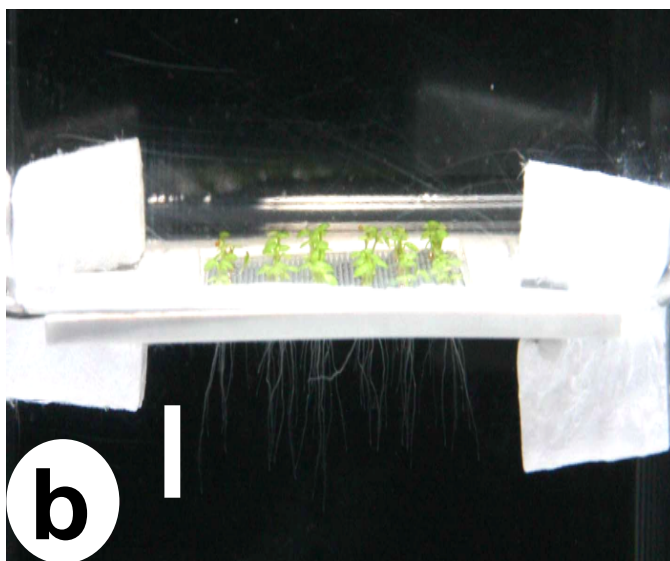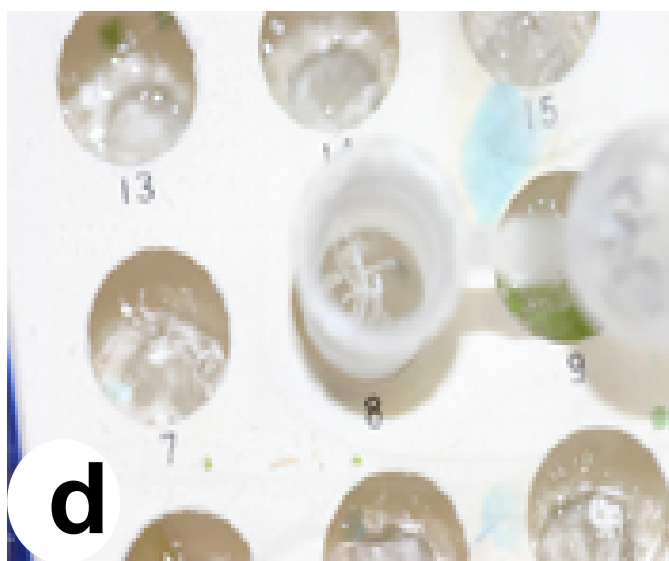**B****FDA****PI**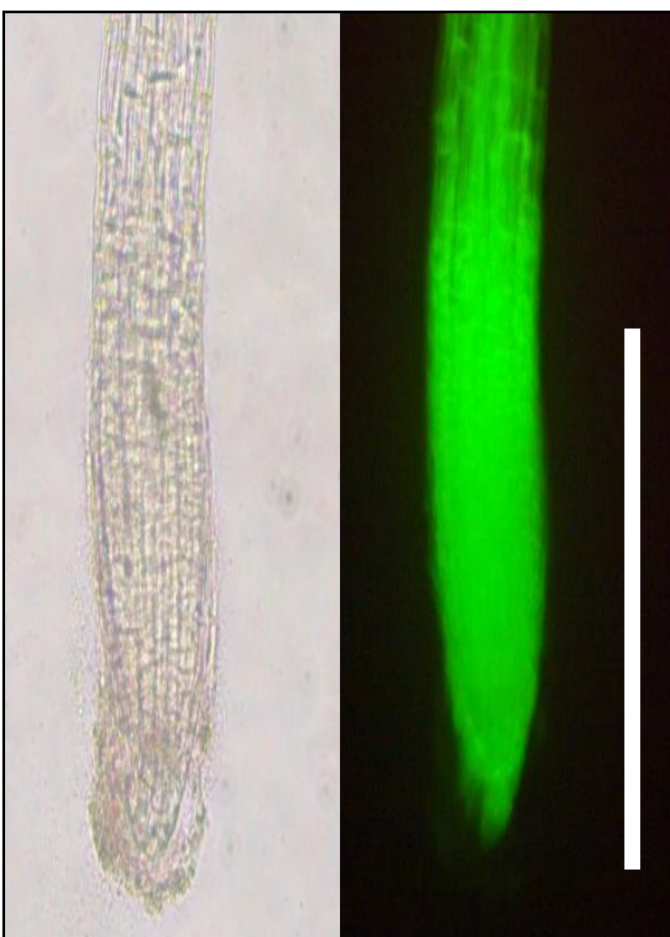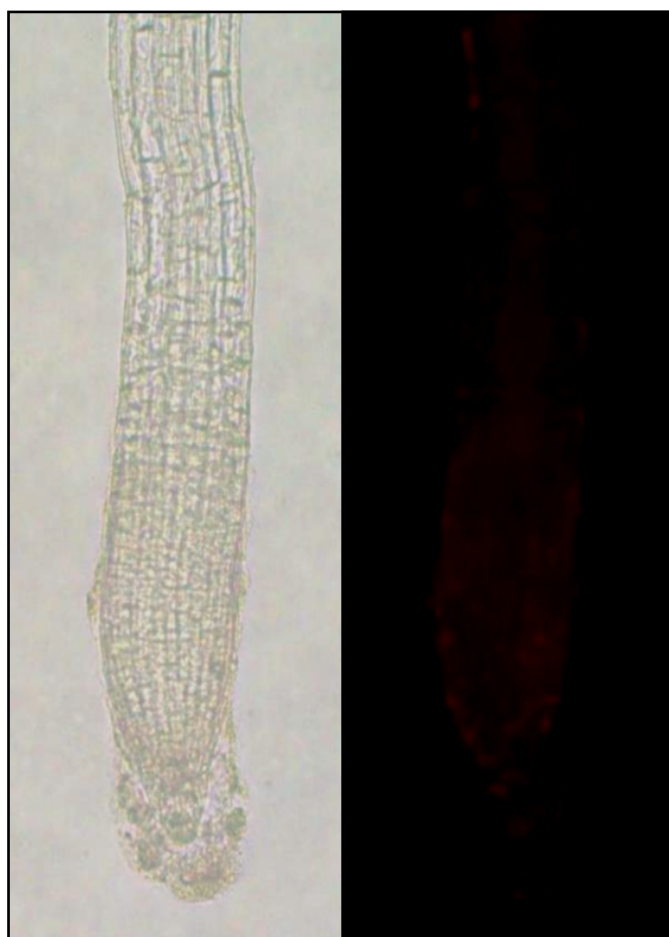

Supplement: Additional file 1 — Hydroponic culture and sampling of root tissues of Arabidopsis. (A) Seedlings were grown on plastic mesh floated on control solution. Top (a) and side (b) views at 10 days are shown. Roots were excised with scissors (c), immediately frozen in liquid nitrogen (d) and used for RNA isolation and microarray analysis. White bar indicates 10 mm. (B) Viability of the root tip in 10-day-old seedlings grown in the culture apparatus in control solution. Root tips were stained with fluorescein diacetate (FDA) and propidium iodide (PI). Bright field images are also shown. White bar indicates 100 μm. [file 1471-2229-9-32-S1.pdf]

**FDA**

**PI**

**Al**

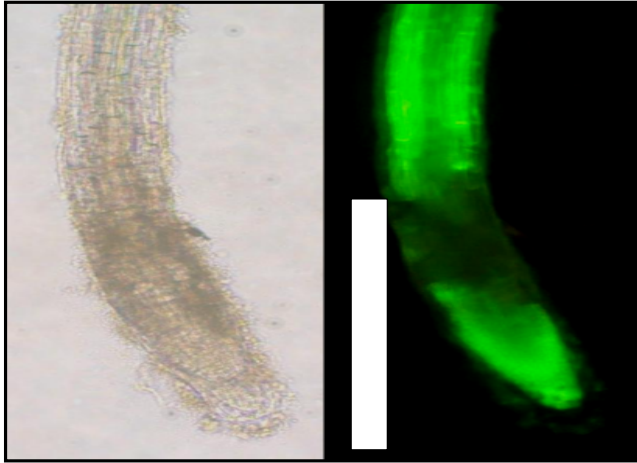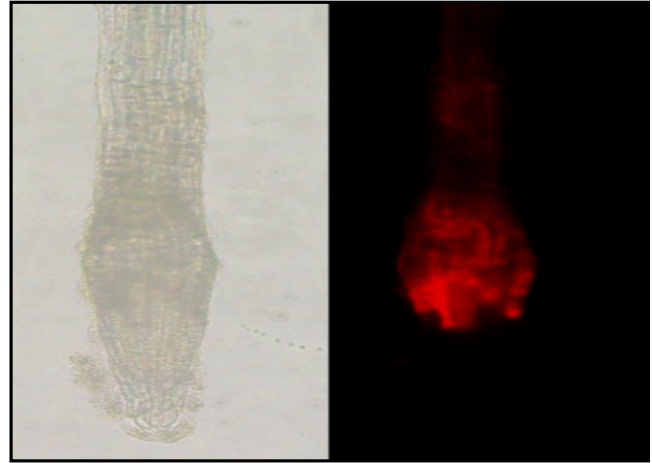

**NaCl**

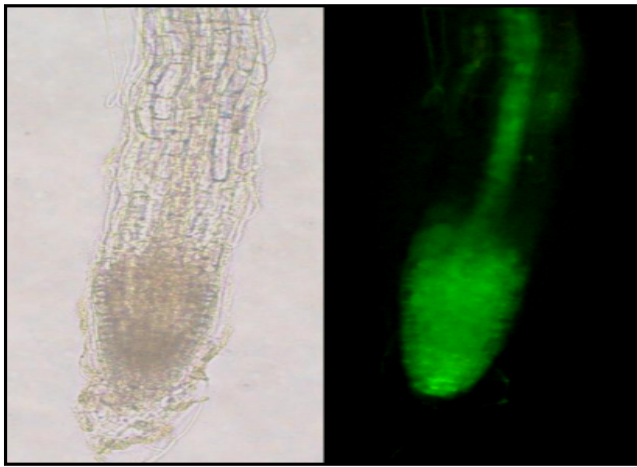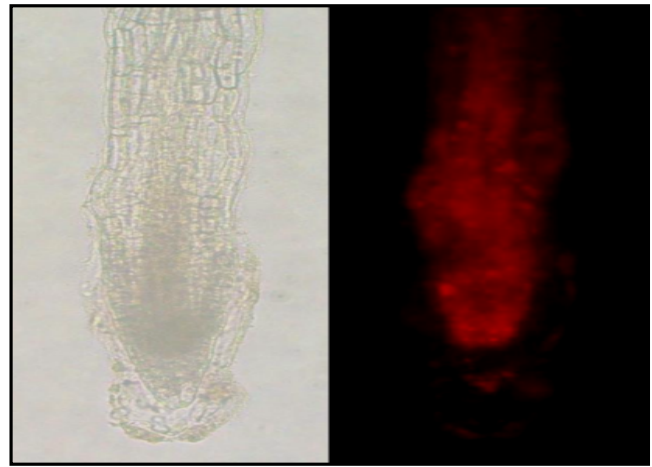

**Cd**

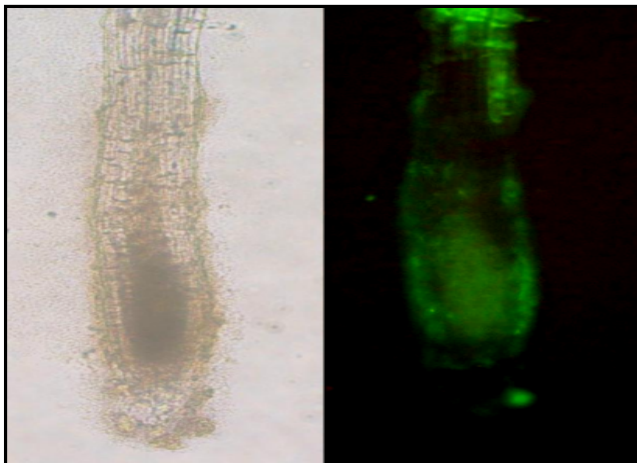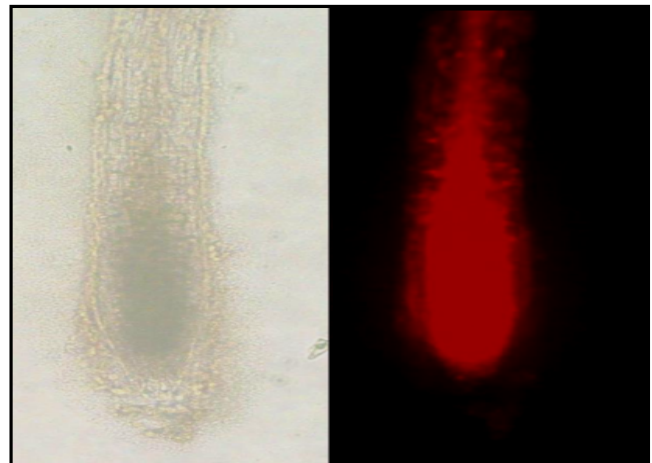

**Cu**

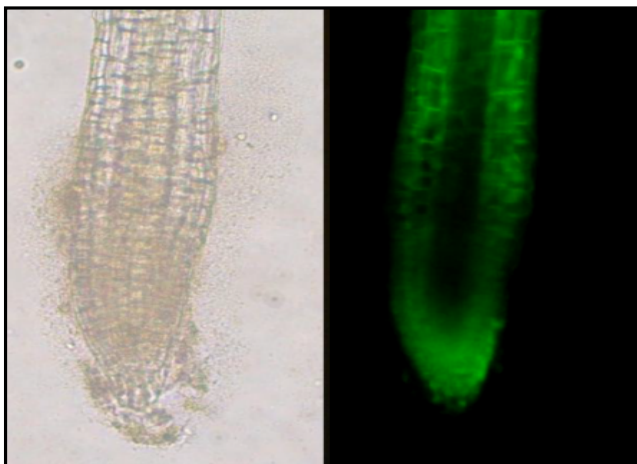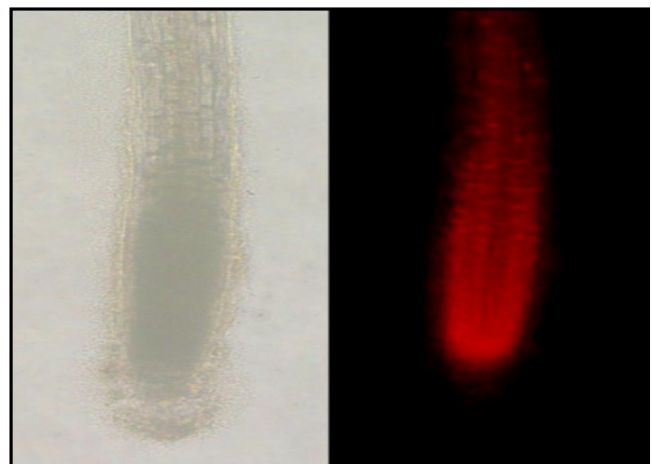

Supplement: Additional file 2 — Viability of root tips of Arabidopsis thaliana under microarray conditions. Seedlings were incubated for 24 h in rhizotoxic solutions (I90 level) and then stained with fluorescein diacetate (FDA) and propidium iodide (PI). Bar indicates 100 μm. Red color indicates damage of the plasma membrane due to PI fluorescence, while green fluorescence of FDA visualizes viable cells. [file 1471-2229-9-32-S2.pdf]

**A**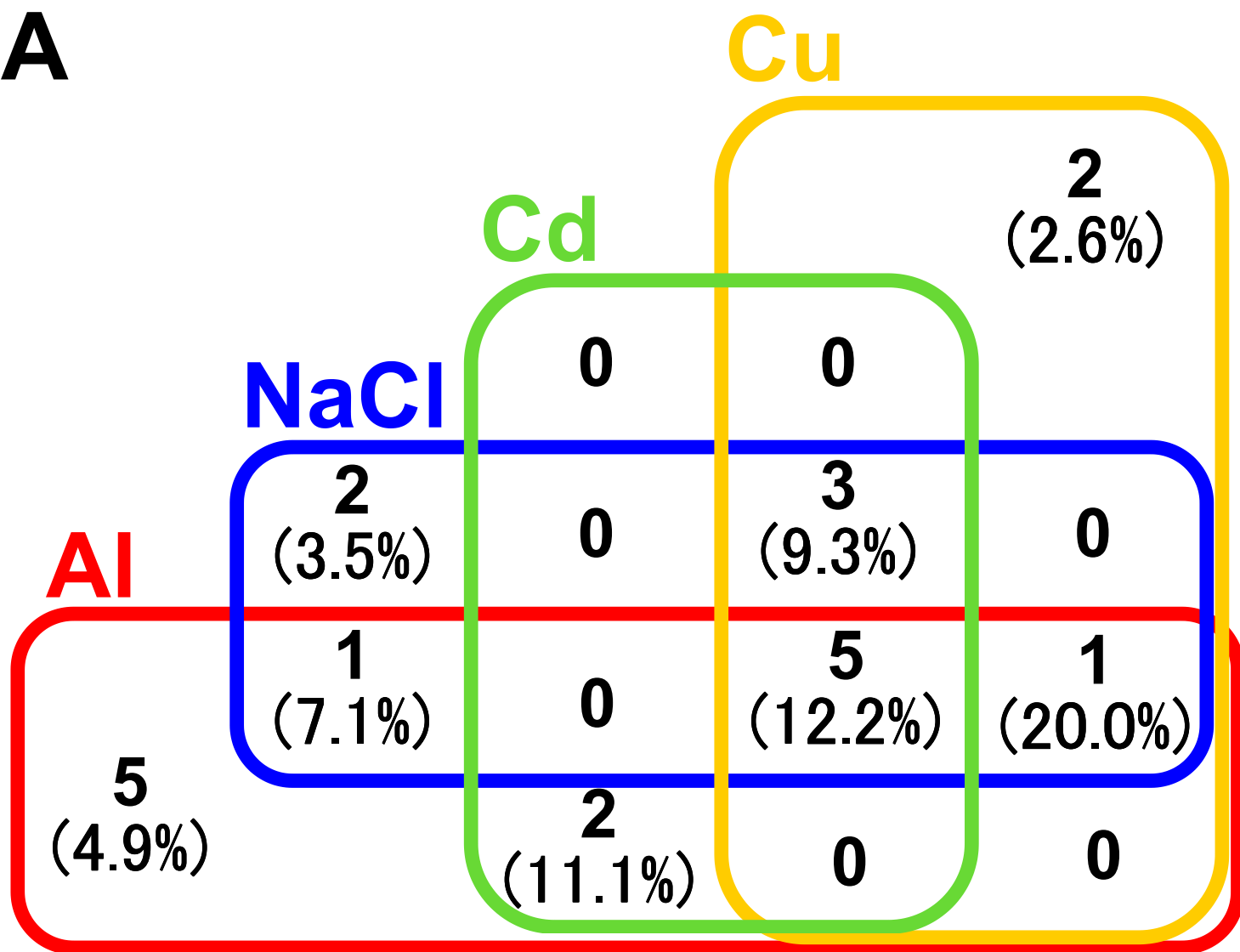**B**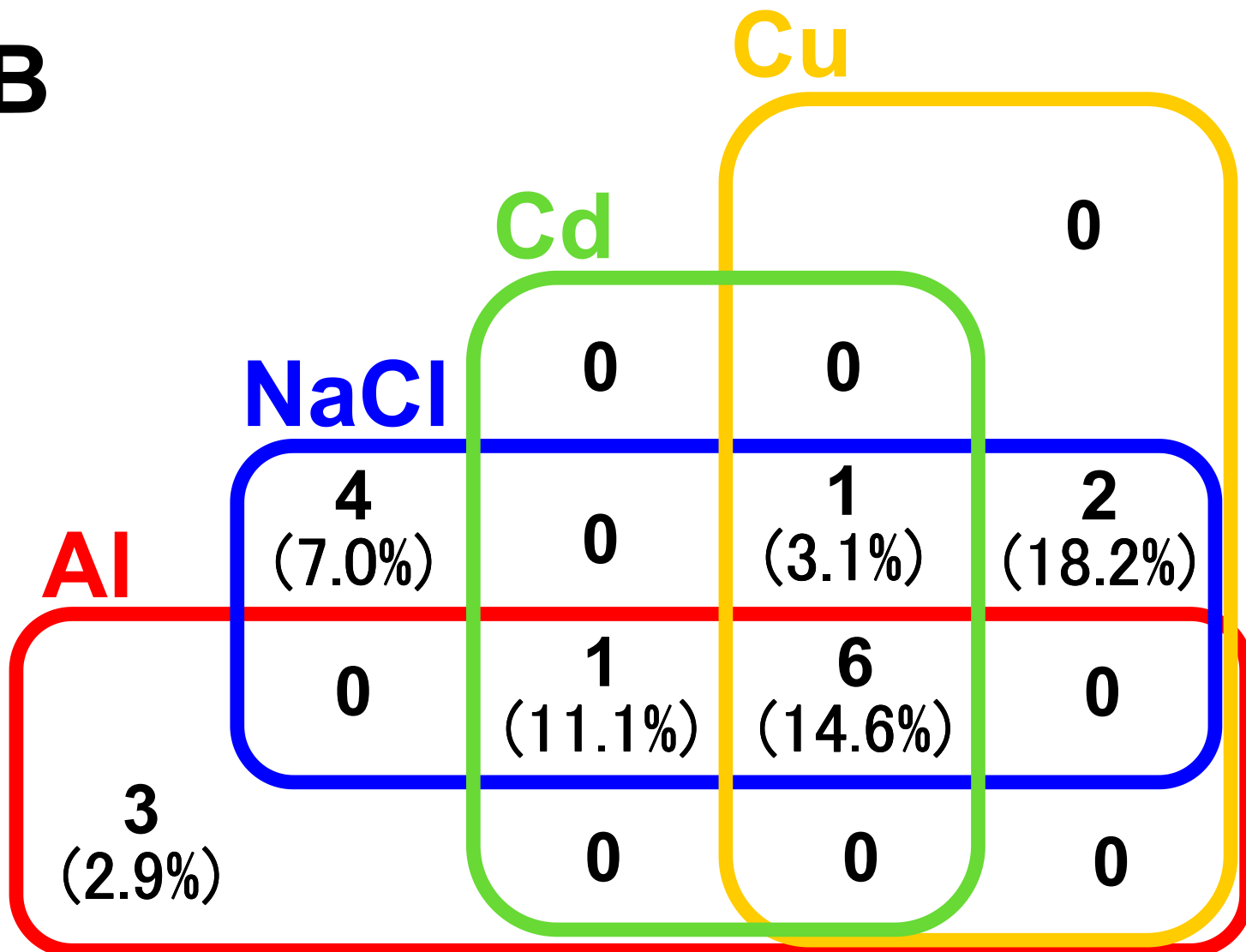

Supplement: Additional file 4 — Grouping of genes encoding ROS-scavenging enzymes and Ca-related proteins among highly inducible genes (i.e. genes grouped in Figure 3) using a Venn diagram approach. (A) Genes encoding ROS-scavenging enzymes, superoxide dismutase, glutathione transferase and peroxidases. (B) Genes encoding proteins carrying "Ca-binding" or "Calmodulin" in their annotation. Relative values (% in each category in Figure 3) are also shown. [file 1471-2229-9-32-S4.pdf]

**H<sub>2</sub>DCFDA****DHE****Cont**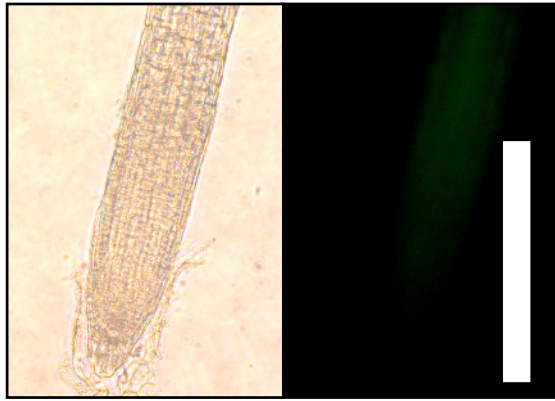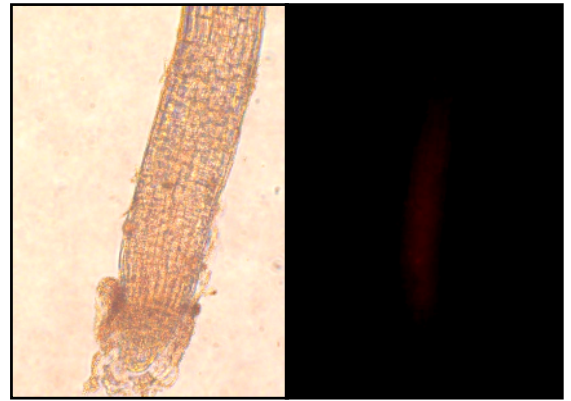**Al**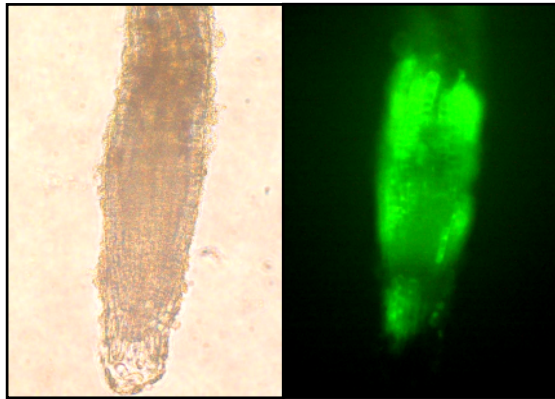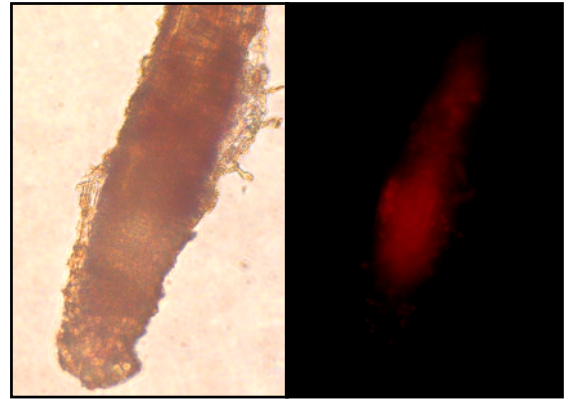**NaCl**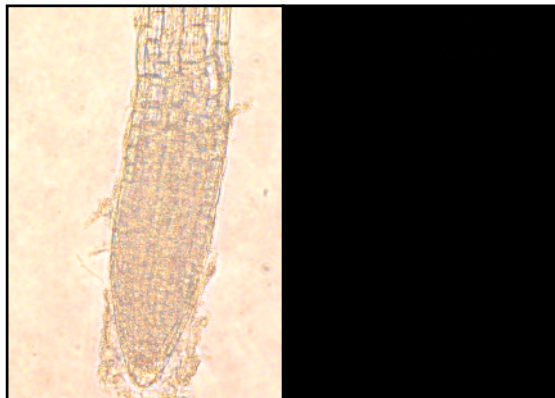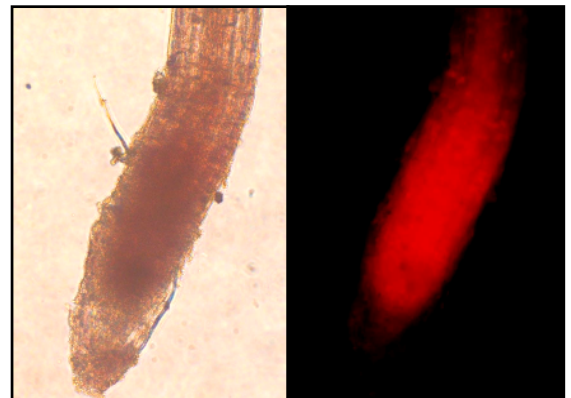**Cd**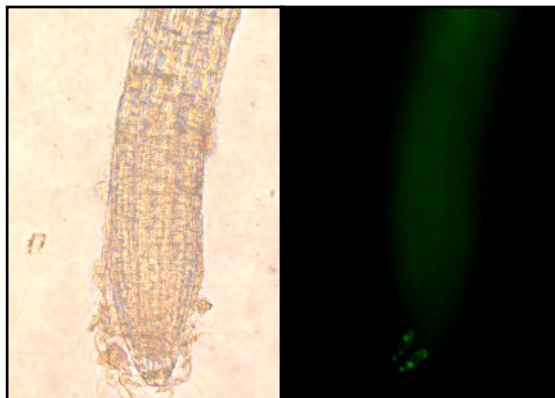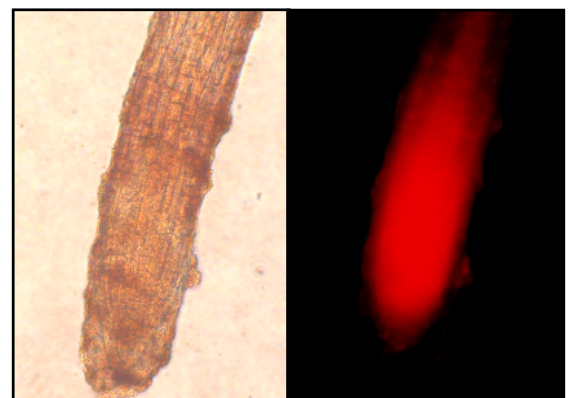**Cu**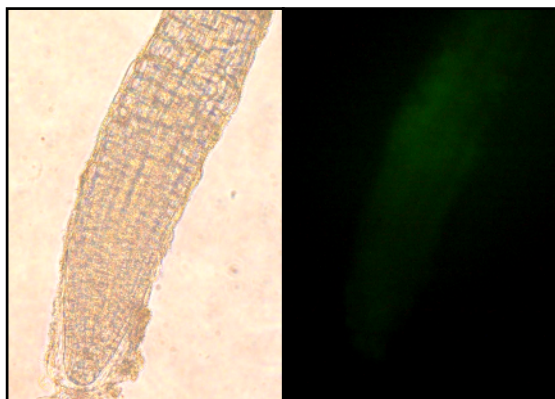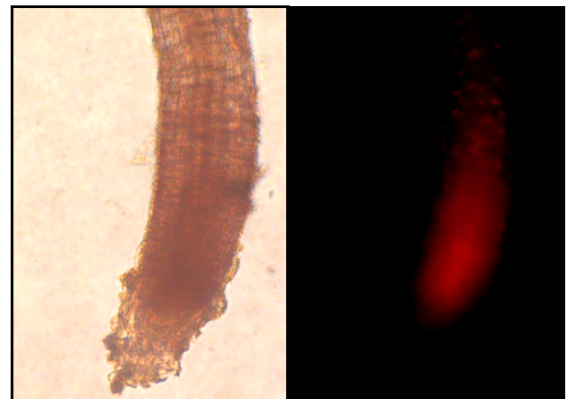

Supplement: Additional file 7 — Histochemical analyses of roots of Arabidopsis thaliana after incubation in rhizotoxic solutions (I50). Growing roots were immersed in rhizotoxic solutions containing AlCl3 (6 μM), NaCl (10 mM), CdCl2 (3 μM) or CuSO4 (1.4 μM) for 24 h, stained with 2',7'-dichlorodihydrofluorescein diacetate (H2DCFDA) or dihydroethidium (DHE), and then observed under a fluorescence microscope. Fluorescent and bright field images are shown. Images of non-stressed roots are shown as controls. White bar indicates 100 μm. [file 1471-2229-9-32-S7.pdf]

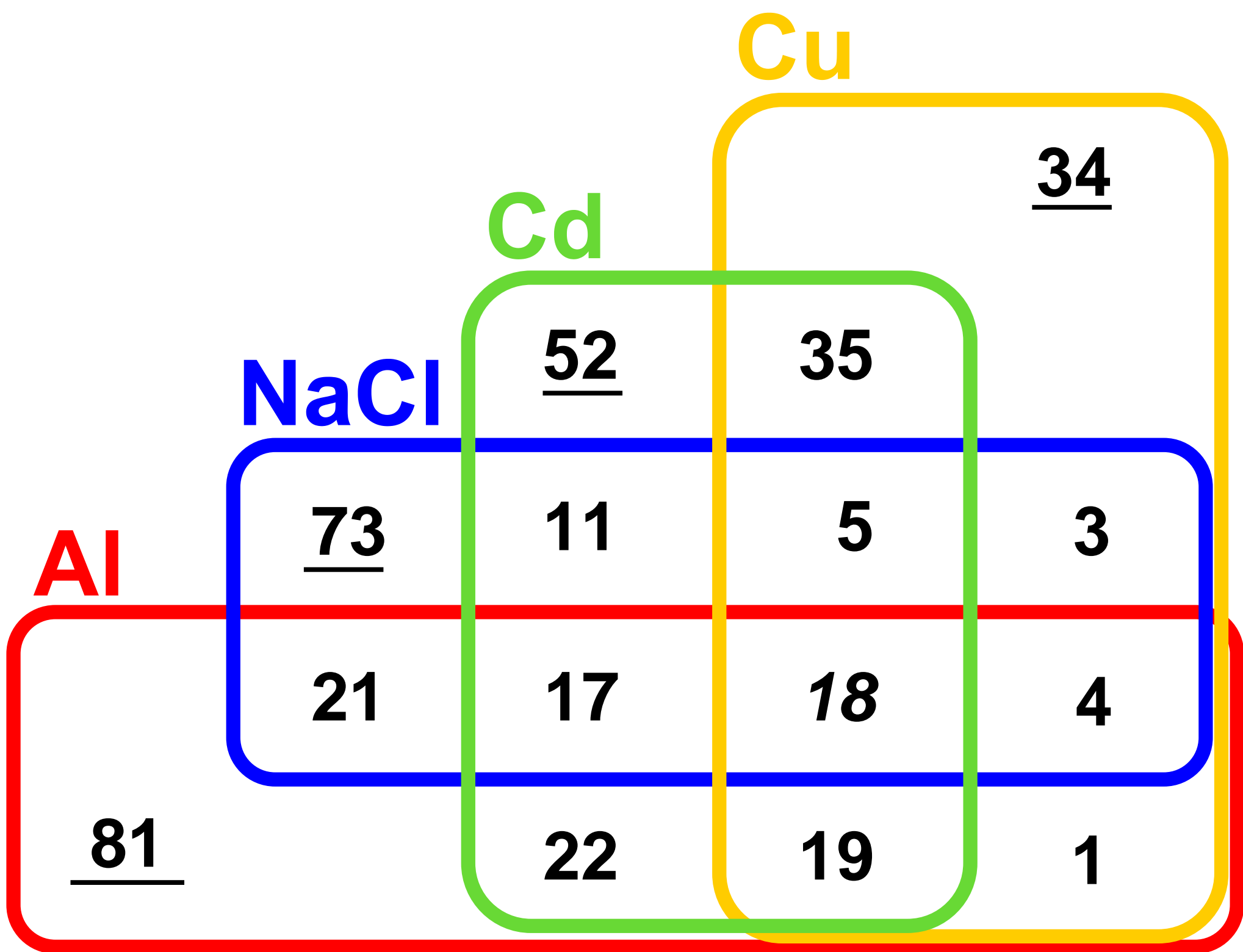

Supplement: Additional file 9 — Venn diagram showing the classification of genes highly downregulated by rhizotoxic ions in Arabidopsis roots. Genes were selected if the fold change value was in the lower 2.5% of quality-controlled spots in each microarray experiment after 24 h incubation with AlCl3 (25 μM), NaCl (50 mM), CdCl2 (15 μM) or CuSO4 (1.6 μM). Genes downregulated in three independent replications were defined as highly downregulated. Genes highly downregulated by each stressor were grouped by Venn diagram. Underlined gene groups consisting of 81 (Al), 73 (NaCl), 52 (Cd) and 34 (Cu) genes were unique for each stressor, while the gene group consisting of 18 genes (italicized) was overlapped by all stressors. [file 1471-2229-9-32-S9.pdf]

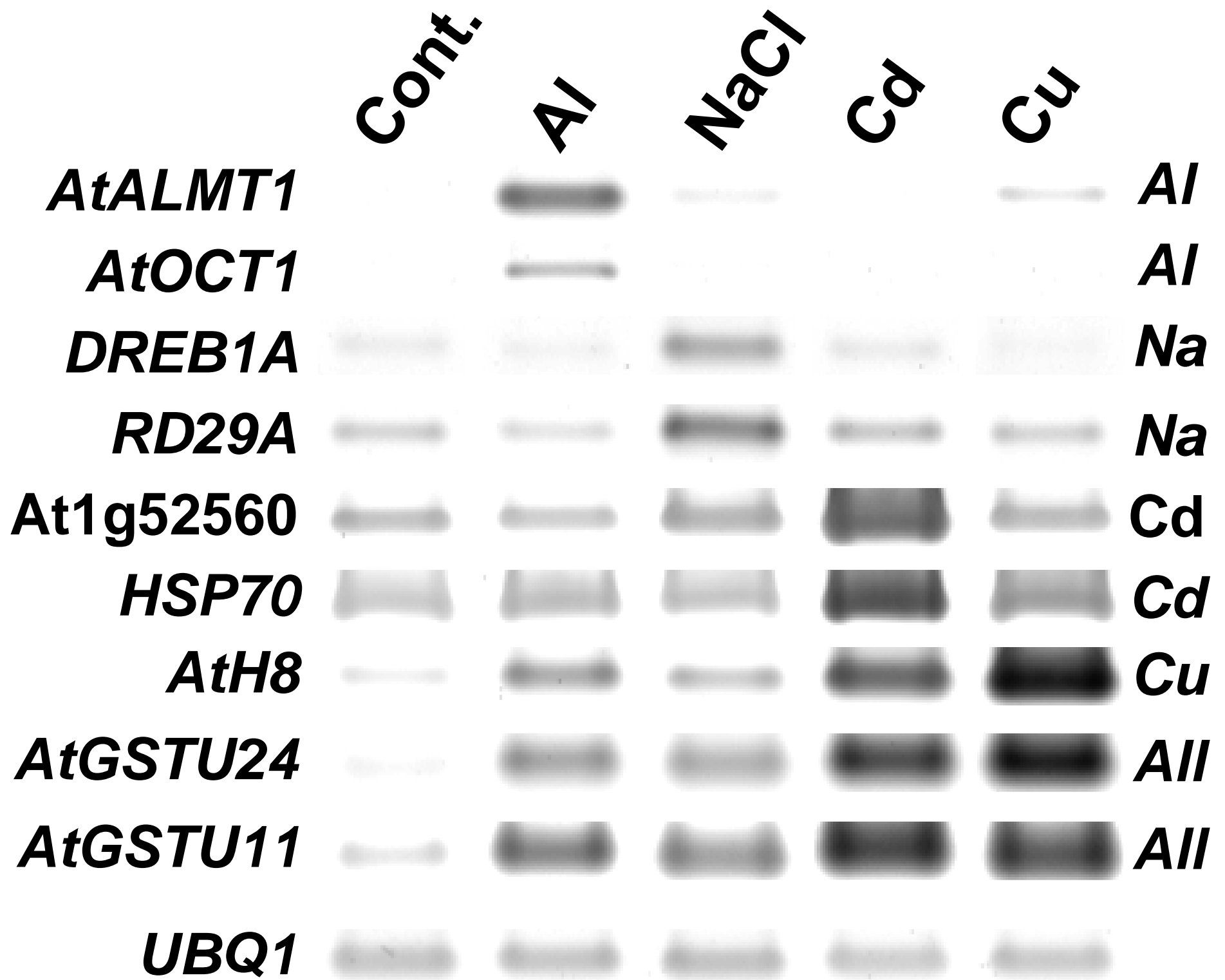

Supplement: Additional file 11 — Gel image of amplicons derived from semi-quantitative RT-PCR for selected genes. Pattern of gene expression profiles of selected genes by Venn diagram (see Figure 3) and cluster analysis (see Figure 4) were analyzed by semi-quantitative RT-PCR. The PCR conditions were optimized to ensure the linear phase of amplification and gel image detection. The UBQ1 expression is shown as control of gene expression. Amplicons were separated on a 3% agarose gel and then visualized with the 1×SYBR Green I (Invitrogen, USA). The gel images were captured with an image analysis system (Typhoon 9400, Amersham Biosciences). Total RNA was isolated from biologically independent root samples with the same rhizotoxic treatments (Al, Cd, Cu ions and NaCl) that were used for the microarray analysis. PCR condition and primers were as follows: AtALMT1 forward: 5'-GGC CGA CCG TGC TAT ACG AG-3', reverse: 5'-CTG AAG ATG CCC ATT ACT TA-3'(263 bp, 22 cycles); AtOCT1 forward: 5'-TTTCTTGTGGCTGTTCCTTCCACAC-3', reverse: 5'-TCT GGA ATT GGA TCG ACT AGG CTT A-3'(548 bp, 23 cycles); DREB1A forward: 5'-GAT GTG TGA TGC GAC GAC G-3', reverse: 5'-TCC ACT GTA CGG ACG GAA G-3'(182 bp, 26 cycles); RD29A forward: 5'-TTC AGA CTA TCT TAG TGG T-3', reverse: 5'-CGT CAC CAA AGC CCA CCG G-3'(281 bp, 26 cycles); At1g52560 forward: 5'-ATA CGA GGT TCC AGG GCT AAC CAA A-3', reverse: 5'-CAA AAA CGA CAC CGT ATC TCT TCT A-3'(305 bp, 31 cycles); HSP70 forward: 5'-TGT ACC AAG GAG CTG GGC CTG ATA T-3', reverse: 5'-GCC CAG TCG TCT TTC ATA GGT CAG A-3'(275 bp, 31 cycles); AtH8 forward: 5'-AGG CTC AAC GCT CTT AAA GAC ACC A-3', reverse: 5'-TGA ATA CAA TCG CAG GTA AAG TGC T-3'(205 bp, 28 cycles); AtGSTU24 forward: 5'-TCA TTA CAT TCA TTT CCG AAC GTA G-3', reverse: 5'-TTA TTA TGC ATT ACA TAG ACC TCA A-3'(119 bp, 25 cycles); AtGSTU11 forward: 5'-TAT CGA AAA ACT GGT CCA GTT CGC T-3', reverse: 5'-CCT TTT AAC TAA ACG AGT TTA CAT C-3'(150 bp, 33 cycles). [file 1471-2229-9-32-S11.pdf]
